# Phase Equilibria, Thermodynamics and Solidified Microstructure in the Copper–Zirconium–Yttrium System

**DOI:** 10.3390/ma16052063

**Published:** 2023-03-02

**Authors:** Fengting Jing, Yuling Liu, Yong Du, Chenying Shi, Biao Hu, Xiancong He

**Affiliations:** 1 State Key Laboratory of Powder Metallurgy, Central South University, Changsha 410083, China; 2 School of Materials Science and Engineering, Anhui University of Science and Technology, Huainan 232001, China; 3 School of Material Science and Engineering, Nanjing Institute of Technology, Nanjing 211167, China

**Keywords:** Cu–Zr–Y system, phase diagram, liquidus projection, CALPHAD, solidified microstructures

## Abstract

A copper alloy with the addition of zirconium and yttrium is an attractive high strength and high conductivity (HSHC) copper alloy. The study of the solidified microstructure, thermodynamics and phase equilibria in the ternary Cu–Zr–Y system is expected to provide new insight into designing an HSHC copper alloy. In this work, the solidified and equilibrium microstructure and phase transition temperatures in the Cu–Zr–Y ternary system were studied by X-ray diffraction (XRD), electron probe microanalysis (EPMA) and differential scanning calorimeter (DSC). The isothermal section at 973 K was experimentally constructed. No ternary compound was found, while the Cu_6_Y, Cu_4_Y, Cu_7_Y_2_, Cu_5_Zr, Cu_51_Zr_14_ and CuZr phases substantially extended into the ternary system. According to the experimental phase diagram data from the present work and the literature, the Cu–Zr–Y ternary system was assessed using the CALPHAD (CALculation of PHAse diagrams) method. The isothermal sections, vertical section and liquidus projection calculated by the present thermodynamic description agree well with the experimental results. This study not only establishes a thermodynamic description of the Cu–Zr–Y system, but also contributes to the design of a copper alloy with the required microstructure.

## 1. Introduction

Copper alloy has excellent electrical and thermal conductivity, good ductility and moderate strength. It is widely used in new energy, electrical and electronics, rail transit and so on [1,2,3]. With the continuous iterative development of products, the requirements for the properties of copper alloy are gradually increasing [4,5,6,7,8]. The high strength and high conductivity (HSHC) copper alloy is drawing growing interest. The influence of zirconium (Zr) on the properties of copper alloy has been systematically studied by many researchers [9,10,11]. Peng et al. [9] studied phase transition for the Cu-0.12 wt.% Zr alloy in the course of aging at 723 K and concluded that the Cu_5_Zr precipitation contributes to the strength. Du et al. [10] investigated the Zr-containing precipitate evolution of the copper–chromium–zirconium alloy and noted that the yield strength was mainly influenced by the Cu_5_Zr phase, Cr-rich precipitation and Zr-rich atomic clusters. To further improve the performance of copper alloy, more and more studies [12,13,14,15,16] have been conducted on the microstructure and properties influenced by the rare earth elements added to copper alloy. The properties for the copper–zirconium–yttrium alloy after cold-rolling and aging were studied systematically by Gao et al. [12] using selected area electron diffraction (SAED) and transmission electron microscopy (TEM), and they concluded that second-phase precipitation can be promoted by adding yttrium (Y). Wang et al. [15] noted that the addition of Y into a Cu–Cr alloy will inhibit the growth of Cr precipitation and decrease the dislocation density, thus increasing the ultimate tensile strength and hardness after cold-rolling and aging. As depicted above, the addition of Zr and Y can promote strength during aging in copper alloy, and Cu–Zr–Y alloy is a key system for potential HSHC copper alloy. The aging process is driven by thermodynamics and knowledge of phase equilibrium and thermodynamics is essential to design compositions and optimize the aging process [17]. Therefore, obtaining an accurate thermodynamic description for the Cu–Zr–Y ternary system is expected to provide new insight into designing an HSHC copper alloy.

Thus far, only two studies on the phase equilibria of the Cu–Zr–Y system have been reported. He et al. [18] first determined the partial isothermal section at 978 K by an electron probe microanalysis (EPMA) technique applied to diffusion couples and extrapolated the ternary isothermal section according to the thermodynamic parameters of three binary systems. No ternary compound was found, and the solubilities of Y in the Cu–Zr system and those of Zr in the Cu–Y system were not considered. The crack in the diffusion-triple affected the results and eight three-phase regions were estimated in the Cu–Zr–Y system by alloy-sampling and diffusion-triple. He et al. [18] noted that measuring three-phase equilibrium was difficult due to electron scattering effects and the presence of a lot of small phase regions around the tri-junction points. Myronenko et al. [19] studied the isothermal section of the Cu–Zr–Y system at 870 K by the alloy annealed at 870 k for 720 h with X-ray diffraction (XRD) and microstructural analysis. The solubility of Y in the Cu_51_Zr_14_ phase was surprisingly high compared to Cu_51_Zr_2.3_Y_11.7_, but no detailed experimental data were provided. The solubilities of Y in the Cu–Zr system and those of Zr in the Cu–Y system are still not clearly characterized.

Further experimental studies for the Cu–Zr–Y system are therefore warranted to provide basic information for the thermodynamic description. The purposes of this work are (1) to experimentally investigate the solidification and equilibrium microstructure and phase transition temperatures in the Cu–Zr–Y system; (2) to evaluate experimental data from the present work and literature to optimize thermodynamic parameters; and (3) to calculate the phase diagram and perform the Scheil solidification simulations.

## 2. Experimental Procedure

The purities of the elements used were Cu-99.99 wt.%, Zr-99.95 wt.% and Y-99.99 wt.%. The alloys were prepared in an arc furnace (WKDHL-1, Opto-electronics Co. Ltd., Beijing, China) with a water-cooled copper crucible in a highly pure argon environment. The alloys were flipped and remelted at least four times to ensure uniformity. The weight of each sample was usually between 4–6 g. The weight loss of every alloy was lower than 2.5%. The real overall composition of the alloys was determined through inductively coupled plasma-optical emission spectrometry (ICP-OES, Thermo Fisher Scientific Inc., Waltham, MA, USA). Each alloy was cut into two parts using wire electrical discharge machining (EDM). One part was analyzed as an as-cast alloy. The other part was encapsulated in a vacuum quartz pipe. A total of 12 samples were melted and numbered sequentially as A1–A12, and their compositions are listed in Table 1. Alloys A1–A8 were annealed at 973 K for 40 days, then water quenched. Alloys A9–A12 were annealed at 1073 K for 40 h, and then annealed at 973 K for 30 days and finally water quenched. XRD (Bruker-AXS D8) and EPMA with wavelength dispersive X-ray spectroscopy (WDX) (JXA-8230, JEOL, Japan) were then used to determine the microstructure and components of these alloys. Afterwards, differential scanning calorimeter (DSC, Netzsch, Germany) measurements of the phase change temperatures of the annealed alloys were performed. The DSC device was performed in an Al_2_O_3_ crucible under a continuous flow of argon (99.998 wt.% purity) with a heating rate of 5 K/min from 303 K to 1323 K. The temperatures for the invariant reactions and liquidus were defined by the initial and peak temperatures, respectively.

## 3. Thermodynamic Models

**Figure 1:**
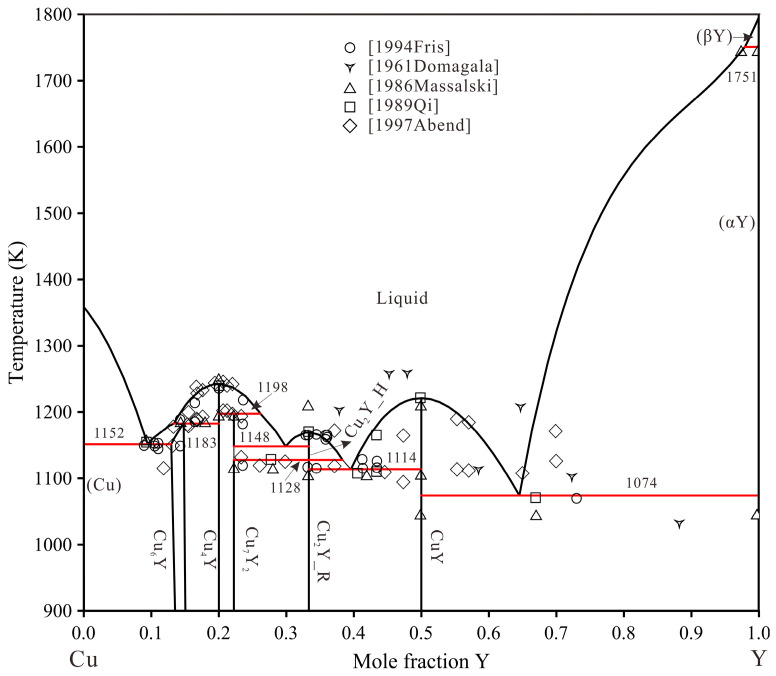
Calculated Cu–Y phase diagram using the parameter from Fries et al. [20] and this work.

**Figure 2:**
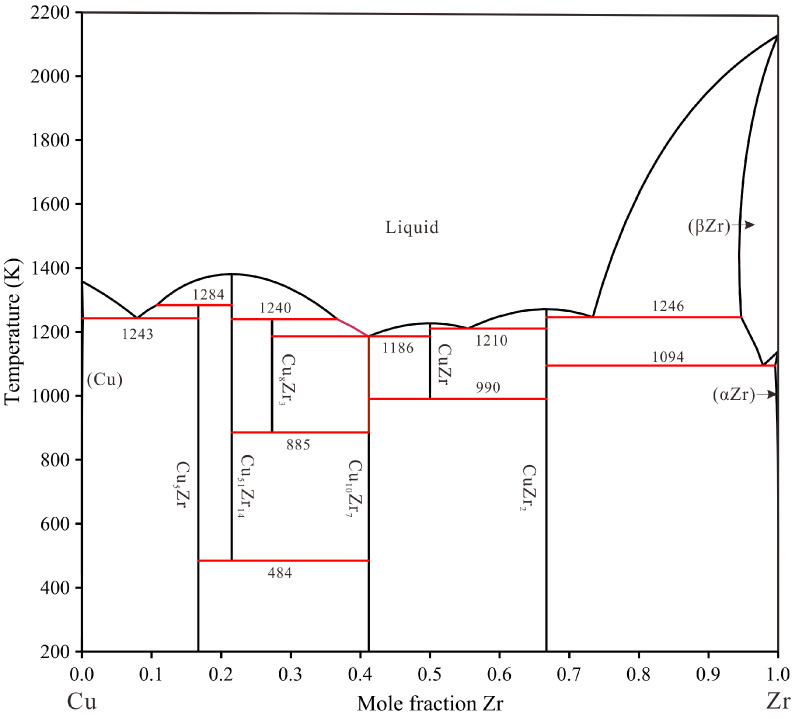
Calculated Cu–Zr phase diagram using the parameter from Liu et al. [21].

**Figure 3:**
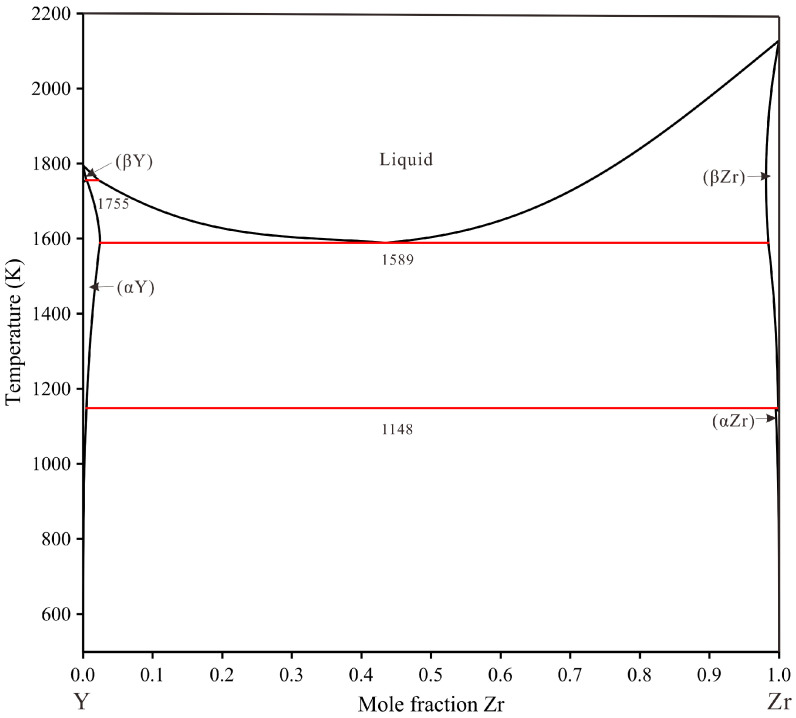
Calculated Y–Zr phase diagram using the parameter from Bu et al. [22].

The phase diagram of the Cu–Y system was minutely adjusted in the present work based on the work of Fries et al. [20]. The phase diagrams of the Cu–Zr system by Liu et al. [21] and the Zr–Y system by Bu et al. [22] are accepted in this work due to the consistency of the thermodynamic databases of copper alloy [23,24,25,26]. The crystallographic data for stable phases in the Cu–Zr–Y system are shown in Table S1 [20,27,28,29,30,31,32,33,34,35] in the supporting information. The calculated binary sub-systems phase diagrams are shown in Figure 1, Figure 2 and Figure 3.

### 3.1. Pure Elements

The Gibbs energy function for the pure element i (i=Cu, Zr and Y) is taken from the Scientific Group Thermodata Europe (SGTE) by Dinsdale [36] and described in the form of:

(1) $$G_{i}(T)-H_{i}^{SER}=a+bT+cT\cdot \ln T+dT^{2}+eT^{-1}+fT^{3}+iT^{7}+jT^{-9}$$

where $H_{i}^{SER}$ means the mole enthalpy of element i relative to the stable element reference (SER) at 298.15 K and 1 bar, and T is the absolute temperature in Kelvin.

### 3.2. The Solution Phases

In the Cu–Y and Cu–Zr–Y systems, (Cu) is the fcc phase, (αZr) and (αY) are the hcp phases (βZr) and (βY) are the bcc phases. The liquid, fcc, hcp and bcc phases serve as a substitutional solution model. The molar Gibbs free energy of the solution phase is described as follows:

(2) Gφ0−HSER=∑ixiGiφ0+RT∑ixiln(xi)+Gmφex

where $x_{i}$ is the mole fraction of element i (i=Cu, Zr and Y); Giφ0 denotes the molar Gibbs energy of component i in the state phase φ; and R represents the gas constant. Gmφex represents the excess Gibbs energy, which is described by the Redlich–Kister (R–K) [37] polynomial. In the Cu–Zr–Y system, Gmφex is represented as follows.

(3) Gmφex=xCuxY∑m=0, 1…LCu,Yφm(xCu−xY)m+xCuxZr∑m=0, 1…LCu,Yφm(xCu−xZr)m+xYxZr∑m=0, 1…LY,Zrφm(xY−xZr)m+xCuxYxZrLCu,Y,Zrφ

where Li,jφm is the mth binary interaction parameter, which can be shown by:

(4) Li,jφm=am+bmT

where $L_{Cu,Y,Zr}^{\varphi }$ is ternary interaction parameter, which can be shown by:

(5) LCu,Y,Zrφ=xCuLCu,Y,Zrφ0+xYLCu,Y,Zrφ1+xZrLCu,Y,Zrφ2

The interaction parameters were optimized in this work based on the available experimental data.

### 3.3. Intermetallic Compounds

There are 11 intermetallic compounds in the system. The Cu_2_Y, CuY, Cu_8_Zr_3_, Cu_10_Zr_7_ and CuZr_2_ phases with ignorable homogeneity are considered as stoichiometric compounds according to the literature and current experimental data, whose Gibbs energy description is given as follows:

(6) GAmBn=mGAHSER0+nGBHSER0+a+bT

where m and n stand for the ratios of stoichiometry and GAHSER0 and GBHSER0 represent the molar Gibbs energy referring to the SER states of *A* and B, respectively.

The Cu_4_Y, Cu_6_Y, Cu_7_Y_2_, Cu_5_Zr, Cu_51_Zr_14_ and CuZr phases with solubilities of a third element were described by a two-sublattice model. Take a phase φ modeled as ${\left(A,\;B\right)}_{p}{\left(A,\;C\right)}_{q}$ for example. The molar Gibbs energy formula is given as Equation (7):

(7) Gmφ=yA′yA″GA:Aφ+yA′yC″GA:Cφ+yB′yA″GB:Aφ+yB′yC″GB:Cφ+RT[p(yA′lnyA′+yB′lnyB′)+q(yA″lnyA″+yC″lnyC″)+yA′yB′(yA″∑iLA,B:Aφi(yA′−yB′)i+yC″∑iLA,B:Cφi(yA′−yB′)i)+yA″yC″(yA′∑iLA:A,Cφi(yA″−yC″)i+yB′∑iLB:A,Cφi(yA″−yC″)i)

where $y_{A}^{\prime }$ and $y_{A}^{\prime \prime }$ are the fraction of the constituent A in the first and second sublattice, respectively. Li represents the ith optimized interaction parameter in this work.

## 4. Results and Discussion

### 4.1. Microstructure and Phase Transition Temperatures Analysis

In this work, twelve alloys were prepared to determine the primary phase and phase equilibria of the Cu–Zr–Y system at 973 K. Table 1 summarizes the results measured by EPMA and XRD, including the primary phase and solidification paths of the as-cast alloy, as well as the equilibria phases of the annealed alloy. In this work, eight primary phases, i.e., (αY), (αZr), CuZr_2_, Cu_51_Zr_14_, Cu_5_Zr, CuY, Cu_7_Y_2_ and Cu_6_Y, were found. The representative alloys are discussed in detail below. Table 2 lists the temperatures of the invariant reactions and liquidus measured by DSC.

#### 4.1.1. Microstructure of Solidification

**Figure 4:**
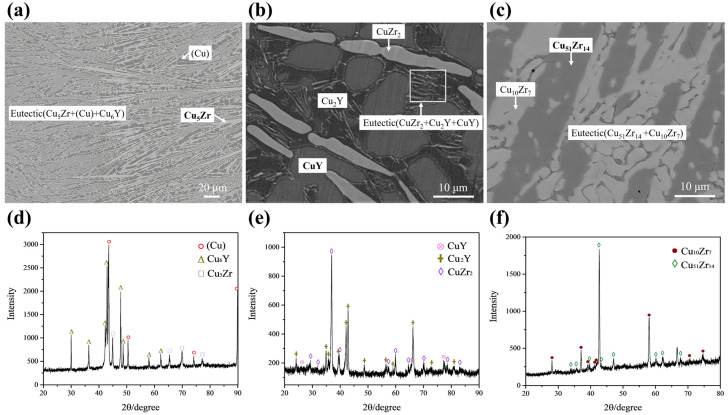
BSE micrographs and XRD patterns of as-cast alloys: (**a**,**d**) A1 Cu_90_Zr_8_Y_2_ (at.%); (**b**,**e**) A9 Cu_53_Zr_12_Y_35_ (at.%); (**c**,**f**) A12 Cu_66_Zr_32_Y_2_ (at.%).

**Figure 5:**
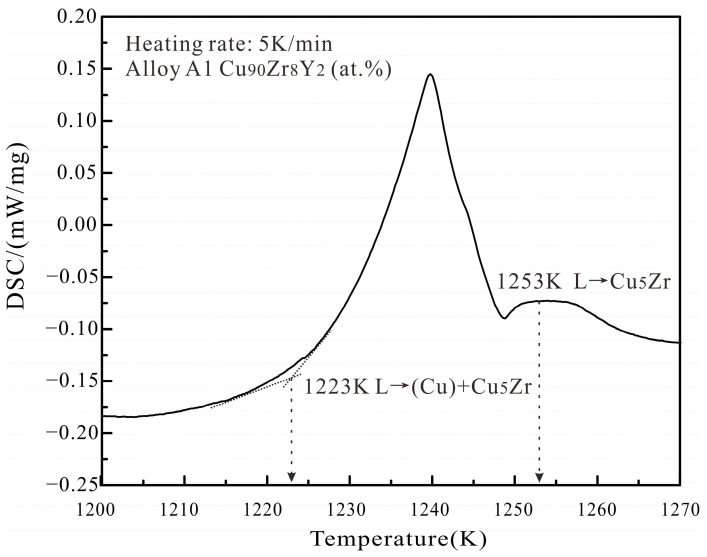
The DSC curves with a heating rate of 5 K/min for annealed alloy A1 Cu_90_Zr_8_Y_2_ (at.%) of the Cu–Zr–Y system.

Figure 4a,d presents the back-scattered electron (BSE) micrograph and XRD pattern of the as-cast alloy A1 Cu_90_Zr_8_Y_2_ (at.%). The primary phase in the images is shown in bold. The analysis shows that the gray phase of Cu_5_Zr is the primary phase, and there are a lot of eutectic structures around it. During the arc-melting process, the side close to the copper crucible cools faster and the solidified microstructure is coarser, while the microstructure further away from crucible is finer. As shown in Figure 4a, the microstructure on the right is coarser than that on the left side. The coarser and finer microstructures in one alloy were also found in our previous Ag–Cr–Zr alloy [24]. Combined with the XRD results, the eutectic structure should be composed of (Cu) + Cu_5_Zr + Cu_6_Y. It can be postulated that the Cu_5_Zr phase solidifies first during the solidification process. Then the liquidus transformation component point soon contacts L → (Cu) + Cu_5_Zr eutectic reaction. Finally, the eutectic equilibrium reaction of L → (Cu) + Cu_5_Zr + Cu_6_Y occurs quickly, therefore a large number of eutectic structures are formed. Figure 5 shows a representative DSC curve for the A1 alloy with a heating rate of 5K/min. There are two visible peaks on this curve. Combined with the previous solidification analysis, the onset temperature of 1123 K of the first peak corresponds to the reaction of L → (Cu) + Cu_5_Zr. The peak temperature of 1153 K of the second peak corresponds to the liquidus temperature of the alloy.

Figure 4b,e presents the BSE micrograph and XRD pattern of the as-cast alloy A9 Cu_53_Zr_12_Y_35_ (at.%). There are bright phase CuZr_2_, gray phase CuY, black phase Cu_2_Y and a eutectic structure of CuZr_2_ + CuY + Cu_2_Y noted in the white rectangle. The solidification paths of A9 are L → CuZr_2_, L → CuZr_2_ + CuZr and L → CuZr_2_ + Cu_2_Y + CuZr.

Figure 4c,f presents the BSE micrograph and XRD pattern of the as-cast alloy A12 Cu_66_Zr_32_Y_2_ (at.%). There are gray phase Cu_10_Zr_7_, dark gray phase Cu_51_Zr_14_ and a eutectic structure of Cu_10_Zr_7_ + Cu_51_Zr_14_ in the alloy. Combined with the as-cast microstructure and XRD results, the primary phase of A12 is Cu_51_Zr_14_, although the composition of Cu_51_Zr_14_ in the as-cast alloy deviates slightly from the equilibrium phase composition shown in Table 1. The solidification paths of A12 are L → Cu_51_Zr_14_ and L → Cu_10_Zr_7_ + Cu_51_Zr_14_.

#### 4.1.2. Microstructure of Annealed Alloys

The experimental results of 12 alloys annealed at 973 K are summarized in Table 1. No ternary compound exists. Four two-phase regions, i.e., (Cu) + Cu_6_Y, (Cu) + Cu_5_Zr, Cu_51_Zr_14_ + Cu_4_Y, Cu_51_Zr_14_ + Cu_10_Zr_7_, and six three-phase regions, i.e., (Cu) + Cu_6_Y +Cu_5_Zr, Cu_10_Zr_7_ + Cu_2_Y + Cu_7_Y_2_, (αY) + (αZr) + CuY, Cu_2_Y + CuZr_2_ + CuZr, Cu_2_Y + CuZr_2_ + CuY, (αZr) + CuZr_2_ + CuY, are determined experimentally. The relationships are discussed as follows.

**Figure 6:**
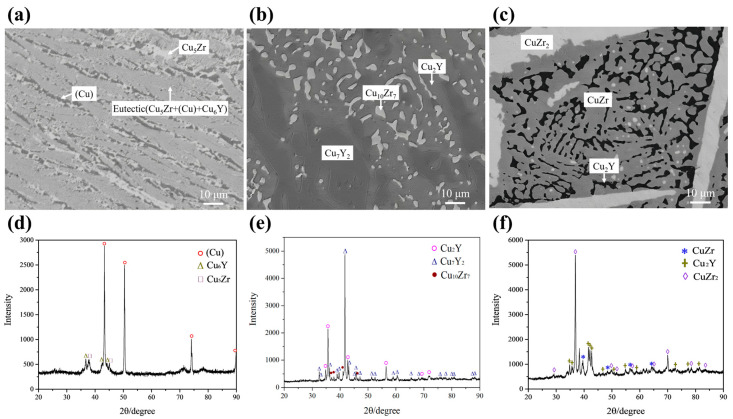
BSE micrographs and XRD patterns of annealed alloys: (**a**,**d**) A1 Cu_90_Zr_8_Y_2_ (at.%); (**b**,**e**) A3 Cu_69_Zr_10_Y_21_ (at.%); (**c**,**f**) A8 Cu_48_Zr_41_Y_11_ (at.%).

Figure 6a,d shows the BSE micrograph and XRD pattern for alloy A1 Cu_90_Zr_8_Y_2_ (at.%), respectively. The annealed microstructure of alloy A1 is similar to that of the cast state shown in Figure 4a. The XRD result indicates the presence of the Cu_6_Y phase and the BSE micrograph illustrates the presence of the Cu_6_Y phase in the eutectic microstructure. Therefore, in conjunction with the analysis above, alloy A1 consists of white Cu_5_Zr, gray Cu_6_Y and black (Cu). The solubility of Y in Cu_5_Zr is 2.03 at.%. The solubility of Zr in Cu_6_Y is 6.61 at.%. Figure 6b,e shows the BSE micrograph and XRD pattern for alloy A3 Cu_69_Zr_10_Y_21_ (at.%), respectively. The annealing alloy A3 is composed of Cu_10_Zr_7_ (white), Cu_2_Y (gray) and Cu_7_Y_2_ (dark). The concave–convex morphology of the Cu_7_Y_2_ phase and Cu_2_Y phase is the same as the result of He et al. [18]. The solubility of Y in Cu_10_Zr_7_ is 1.53 at.%. The solubility of Zr in Cu_7_Y_2_ is 7.83 at.%. Figure 6c,f shows the BSE micrograph and XRD pattern for alloy A8 Cu_48_Zr_41_Y_11_ (at.%), respectively. According to the results, alloy A8 is located in the three-phase region, i.e., bright CuZr_2_, grey CuZr and black Cu_2_Y. The solubility of Y in CuZr is 3.38 at.%.

**Figure 7:**
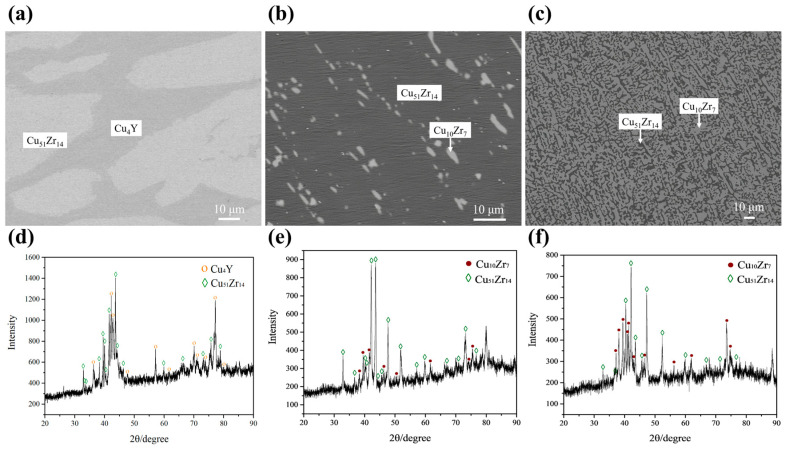
BSE micrographs and XRD patterns of annealed alloys: (**a**,**d**) A7 Cu_79_Zr_13_Y_8_ (at.%); (**b**,**e**) A11 Cu_78_Zr_18_Y_4_ (at.%); (**c**,**f**) A12 Cu_66_Zr_32_Y_2_ (at.%).

Figure 7a,d shows the BSE micrograph and XRD pattern for alloy A7 Cu_79_Zr_13_Y_8_ (at.%), respectively. The results show that alloy A7 consists of light gray Cu_51_Zr_14_ and dark gray Cu_4_Y phases. The A7 alloy is located in the two-phase region of Cu_51_Zr_14_ and Cu_4_Y. Figure 7b,e and Figure 7c,f show the BSE micrographs and XRD patterns for alloys A11 Cu_78_Zr_18_Y_4_ (at.%) and A12 Cu_66_Zr_32_Y_2_ (at.%), respectively. In the standard PDF cards for the Cu_51_Zr_14_ and Cu_10_Zr_7_ phases, there is no standard peak after 77.757° of 2θ, therefore the peak larger than 78° is not calibrated in the XRD pattern. According to the results, alloys A11 and A12 are both located in the grey Cu_10_Zr_7_ and dark gray Cu_51_Zr_14_ two-phase region, although their microstructures differ considerably. More Cu_51_Zr_14_ phases are present in alloy A11. The measured maximum solubility of Y in Cu_51_Zr_14_ is about 4.45 at.%. The solubilities of the 3rd element are determined in this work for the Cu–Zr–Y system. Noticeable solubilities were also measured in the Al–Zr–Y system by Liu et al. [38] and Bao et al. [39].

**Figure 8:**
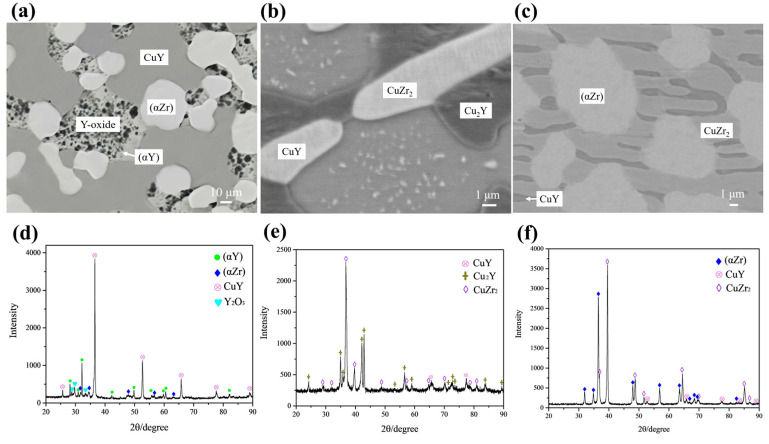
BSE micrographs and XRD patterns of annealed alloys: (**a**,**d**) A4 Cu_33_Zr_7_Y_60_ (at.%); (**b**,**e**) A9 Cu_53_Zr_12_Y_35_ (at.%); (**c**,**f**) A10 Cu_22_Zr_71_Y_7_ (at.%).

Figure 8a,d shows the BSE micrograph and XRD pattern for alloy A4 Cu_33_Zr_7_Y_60_ (at.%), respectively. The results show that alloy A4 is in the three-phase region for white αZr, grey αY and dark CuY. The rare earth Y is chemically active [40]. The oxidation of Y can be observed during the metallographic polishing process, which does not affect the phase equilibrium after vacuum annealing at 973 K. Combined with the XRD result, the black Y-oxide is Y_2_O_3_. The solubility of Cu in the hcp phase is about 2 at.%. Figure 8b,e shows the BSE micrograph and XRD pattern for alloy A9 Cu_53_Zr_12_Y_35_ (at.%), respectively. The diagrams show that alloy A9 is in the three-phase region for white CuZr_2_, grey CuY and black Cu_2_Y. Figure 8c,f shows the BSE micrograph and XRD pattern for alloy A10 Cu_22_Zr_71_Y_7_ (at.%), respectively. The diagrams show that alloy A10 is in the three-phase region for white αZr, grey CuZr_2_ and dark CuY.

**Figure 9:**
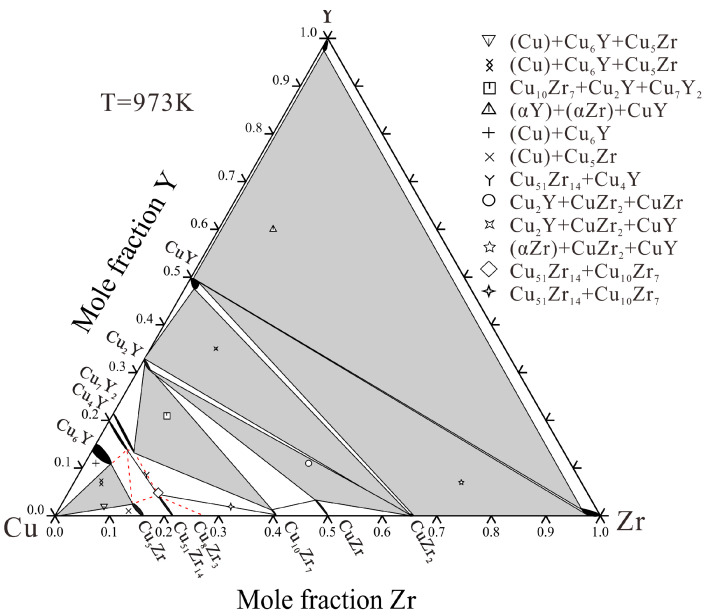
Experimental isothermal section of Cu–Zr–Y system with experimental data at 973 K.

Based on the present experiments, the isothermal section at 973 K is constructed in Figure 9. Three two-phase regions and four three-phase regions agree well with those of He et al. [18]. The red dashed line in the experimental phase diagram indicates the phase regions that were not directly measured by the experiment. The alloys designed in the three-phase region of Cu_4_Y + Cu_6_Y + Cu_5_Zr tend to shift into the three-phase region of (Cu) + Cu_6_Y + Cu_5_Zr due to the small range of the phase region and the easy volatilization of Y during melting. This part has not been experimentally determined.

### 4.2. Thermodynamic Assessment

The PARROT module in Thermo-Calc software [41] was used for optimization based on the least square method. The thermodynamic descriptions for the Cu–Y [20], Cu–Zr [21] and Zr–Y [22] systems in the literature were combined to form a basis for the Cu–Zr–Y system assessment. Before optimizing the ternary system, the thermodynamic parameters of Cu−Y were optimized in order to satisfy the phase relations of (αY) + (αZr) + CuY and (αZr) + CuZr_2_ + CuY at 870K of the Cu–Zr–Y system. After optimization the temperature of the invariant reaction (αZr) + CuY → (αY) + CuZr_2_ was adjusted downward to 860 K. The presently calculated phase diagram of the Cu–Y system is consistent with the experimental data [20,42,43,44] and the calculated date [45], which is shown in Figure 1. For the Cu–Zr–Y system, the experimental data measured by alloy-sampling from the literature [18] are also used in the optimization.

**Figure 10:**
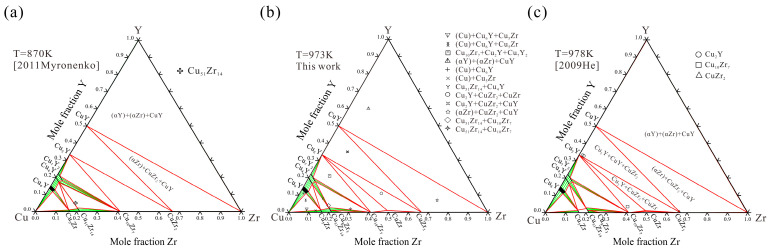
Calculated isothermal sections of Cu–Zr–Y system at (**a**) 870 K with experimental data [19], (**b**) 973 K with experimental data from this work and (**c**) 978 K with experimental data [18].

**Figure 11:**
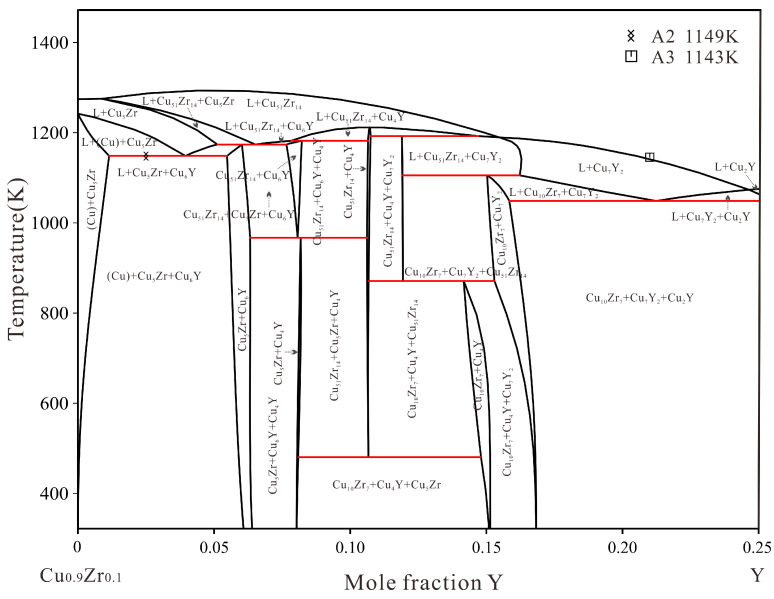
Calculated vertical section of the Cu–Zr–Y system at 10 at.% Zr with the experimental data.

Then the measured ternary solubilities of Cu_6_Y, Cu_4_Y, Cu_7_Y_2_, Cu_5_Zr and Cu_51_Zr_14_ were considered. Optimization was carried out sequentially from the copper-rich end to the copper-poor end. Afterward, all the parameters were optimized simultaneously to achieve reasonable thermodynamic parameters. Finally, Table 3 provides the reasonable parameters of the Cu–Zr–Y system obtained in this work where the adjusted parameters are bolded. According to the current thermodynamic parameters, the calculated isothermal sections at 870 K, 973 K and 978 K are shown in Figure 10 compared with the reported experimental data [18,19] and the present measurement results. These calculations are in good agreement with experimental data. The calculated vertical section of the Cu–Zr–Y system at 10 at.% Zr, compared with the measured temperatures by DSC, are shown in Figure 11. It is noteworthy that only alloy A3 is located at the 10 at.% Zr vertical section. Alloy A2 is labeled in the vertical section by L → (Cu) + Cu_5_Zr + Cu_6_Y invariant reactions.

**Figure 12:**
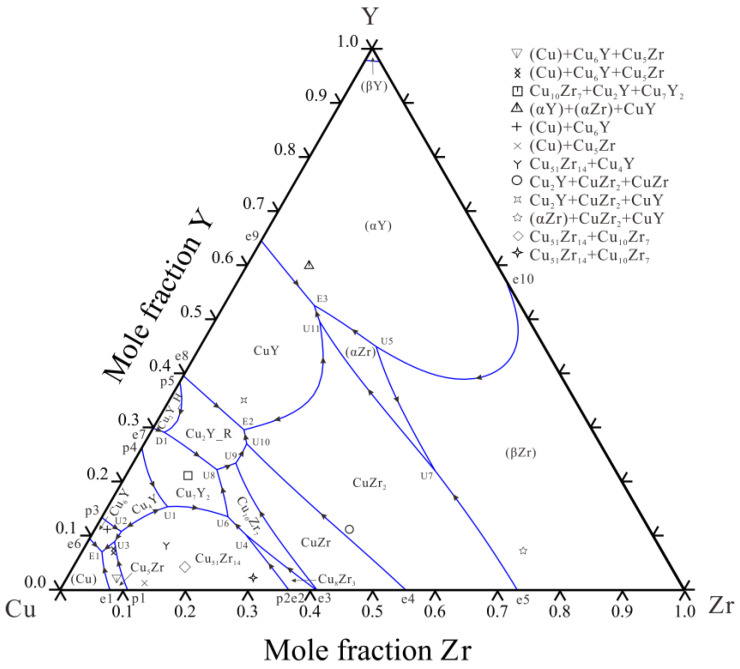
Calculated liquidus projection of the Cu–Zr–Y system with experimental data.

**Figure 13:**
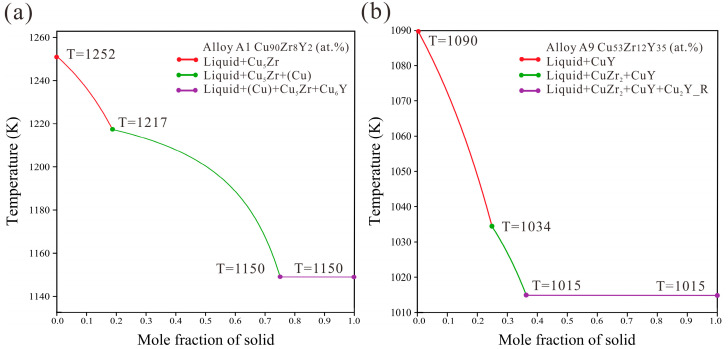
The Scheil simulation for alloys A1 Cu_90_Zr_8_Y_2_ (at.%) (**a**) and A9 Cu_53_Zr_12_Y_35_ (at.%) (**b**).

Figure 12 shows the calculated liquidus projection in comparison with the experimental primary phases. The present thermodynamic parameters are used for Scheil solidification simulation to guide the solidification behavior. Figure 13 shows the Scheil solidification simulation results of alloys A1 and A9. It can be seen that the primary phases of alloys A1 and A9 are Cu_5_Zr and CuY, respectively. The ternary eutectic structure of (Cu) + Cu_5_Zr + Cu_6_Y and CuZr_2_ + Cu_2_Y + CuZr are formed. These Scheil solidification simulations are consistent with the experimental result discussed in Section 4.1.1.

Based on the CALPHAD method and key experiments, a reasonable set of thermodynamic parameters for the Cu–Zr–Y system was obtained. The thermodynamic parameters were used to simulate phase equilibrium and Scheil solidification, which can guide the design of composition and microstructure of HSHC copper alloy.

## 5. Conclusions and Summary

The solidification microstructure, phase equilibria and phase transition temperatures in the Cu–Zr–Y ternary system were investigated by XRD, EPMA and DSC technologies. Furthermore, the thermodynamic optimization and a sequence of calculations and simulations using the present thermodynamic parameters were carried out. The major conclusions are as follows:

- The solid solubility in the ternary system is determined. The maximum solubility of Zr in Cu_6_Y, Cu_4_Y and Cu_7_Y_2_ are about 6.61, 6.27 and 7.83 at.% Zr, respectively. The solubility of Y in Cu_5_Zr, Cu_51_Zr_14_ and CuZr are about 2.57, 4.45 and 3.38 at.% Y, respectively. The solubility of Cu in the hcp phase is about 2 at.%.
- The Cu–Y system and the Cu–Zr–Y system were optimized by the CALPHAD method. The calculated isothermal sections, liquidus projection and vertical section are consistent with the experimental data.
- The observed solidified microstructure agrees with the result of the Scheil solidification simulations using the thermodynamic parameters. The presently obtained thermodynamic description for the Cu–Zr–Y system can be used to guide the composition and microstructure design of Cu–Zr–Y alloys.

## Figures and Tables

**Table 1:** Summary of the experimental data for the Cu–Zr–Y system.

Alloy	Alloy	Primary	Primary Phase				Phase	Equilibrium Phase		
No.	Composition	Phase	Composition (at.%)			Solidification Paths	Equilibria	Composition (at.%)		
	(at.%)		Cu	Y	Zr			Cu	Y	Zr
A1	Cu90Zr8Y2	Cu_5_Zr	83.05	1.05	15.9	L → Cu_5_Zr	(Cu)	99.95	0	0.05
						L → (Cu) + Cu_5_Zr	Cu_5_Zr	83.22	2.03	14.75
						L → (Cu) + Cu_5_Zr + Cu_6_Y	Cu_6_Y	84.69	8.7	6.61
A2	Cu88Zr5Y7	Cu_5_Zr	82.84	7.08	10.08	L → Cu_51_Zr_14_	(Cu)	99.62	0.15	0.23
						L + Cu_51_Zr_14_ → Cu_5_Zr	Cu_5_Zr	82.20	2.57	15.23
						L → (Cu) + Cu_5_Zr	Cu_6_Y	84.26	9.84	5.9
A3	Cu69Zr10Y21	Cu_7_Y_2_	77.18	17.00	5.82	L → Cu_7_Y_2_	Cu_10_Zr_7_	59.74	1.53	38.73
						L → Cu_7_Y + Cu_2_Y	Cu_2_Y	67.59	31.79	0.62
						L + Cu_7_Y_2_ → Cu_10_Zr_7_ + Cu_2_Y	Cu_7_Y_2_	78.79	13.38	7.83
A4	Cu33Zr7Y60	(αY)	4.78	94.28	0.94	L → (αY)	(αY)	2.06	97.26	0.68
						L → (αY) + (αZr)	(αZr)	2.81	1.51	95.68
						L → (αY) + (αZr) + CuY	CuY	49.64	49.76	0.60
A5	Cu87Zr2Y11	Cu_6_Y	84.25	11.12	4.63	L → Cu_6_Y	(Cu)	98.80	1.19	0.01
						L → Cu_6_Y + (Cu)	Cu_6_Y	85.34	11.56	3.1
A6	Cu86Zr13Y1	Cu_51_Zr_14_	79.46	0.13	20.41	L → Cu_51_Zr_14_	(Cu)	98.98	0.16	0.86
						L + Cu_51_Zr_14_ → Cu_5_Zr	Cu_5_Zr	83.07	1.04	15.89
						L → (Cu) + Cu_5_Zr				
A7	Cu79Zr13Y8	Cu_51_Zr_14_	79.17	3.72	17.11	L → Cu_51_Zr_14_	Cu_51_Zr_14_	78.87	3.99	17.14
						L → Cu_4_Y	Cu_4_Y	79.88	13.85	6.27
A8	Cu48Zr41Y11	CuZr_2_	34.52	0.04	65.44	L → CuZr_2_	CuZr_2_	34.55	0.1	65.35
						L → CuZr + CuZr_2_	CuZr	50.54	3.38	46.08
						L + CuZr → Cu_2_Y + CuZr_2_	Cu_2_Y	67.38	30.50	2.12
A9	Cu53Zr12Y35	CuY	50.14	46.08	3.78	L → CuY	CuY	50.76	47.43	1.81
						L → CuY + Cu_2_Y	Cu_2_Y	67.05	32.95	0
						L → CuY + Cu_2_Y + CuZr_2_	CuZr_2_	34.78	0	65.22
A10	Cu22Zr71Y7	(αZr)	4.34	0	95.66	L → (αZr)	(αZr)	2.28	0	97.72
						L → (αZr) + CuZr_2_	CuZr_2_	34.06	0	65.94
						L → (αZr) + CuZr_2_ + CuY	CuY	49.09	48.78	2.13
A11	Cu78Zr18Y4	Cu_51_Zr_14_	79.10	6.16	14.74	L → Cu_51_Zr_14_	Cu_51_Zr_14_	78.80	4.42	16.78
						L → Cu_10_Zr_7_ + Cu_51_Zr_14_	Cu_10_Zr_7_	59.82	0.31	39.87
A12	Cu66Zr32Y2	Cu_51_Zr_14_	73.39	1.87	24.73	L → Cu_51_Zr_14_	Cu_51_Zr_14_	78.23	4.45	17.32
						L → Cu_10_Zr_7_ + Cu_51_Zr_14_	Cu_10_Zr_7_	59.66	0.01	40.33

**Table 2:** Comparison of the DSC results with the calculated results in the Cu–Zr–Y system.

Alloy	Alloy	Phase Transition Temperatures (K)			
No.	Composition	Liquidus		Invariant	
	(at.%)	Exp.	Calc.	Exp.	Calc.
A1	Cu90Zr8Y2	1253	1252		
		1223	1217		
A2	Cu88Zr5Y7	1225	1197	1149	1149
A3	Cu69Zr10Y21	1143	1141		
A4	Cu33Zr7Y60	1093	1090		
A5	Cu87Zr2Y11	1180	1179		
		1153	1151		
A6	Cu86Zr13Y1	1223	1217		
A7	Cu79Zr13Y8	1295	1299		

**Table 3:** The thermodynamic parameters obtained in this work.

Phases	Models	Thermodynamic Parameters
Liquid	(Cu,Y,Zr)_1_	GCu,YLiquid0=− **89300** +21.5669·T
		LCu,Y,ZrLiquid1= **2000**
CuZr	(Cu)_1_(Zr,Y)_1_	GCu:YCuZr0=− **11582** +GCuFcc0+GYHcp0
Cu_51_Zr_14_	(Cu)_51_(Zr,Y)_14_	GCu:YCu51Zr140=− **896480** +51·GCuFcc0+14·GYHcp0
Cu_5_Zr	(Cu)_5_(Zr,Y)_1_	GCu:YCu5Zr0=− **64998** +5·GCuFcc0+GYHcp0
Cu_4_Y	(Cu)_4_(Y,Zr)_1_	GCu:YCu4Y0=− **89500** +8.25205·T+4·GCuFcc0+GZrHcp0
		GCu:ZrCu6Y0=− **40366** +4·GCuFcc0+GZrHcp0
Cu_6_Y	(Cu)_5_(Cu2,Y,Zr)_1_	GCu:YCu6Y0=− **90000** +8.29539·T+5·GCuFcc0+GZrHcp0
		GCu:ZrCu6Y0=− **45447** +5·GCuFcc0+GZrHcp0
Cu_7_Y_2_	(Cu)_7_(Y,Zr)_2_	GCu:YCu7Y20=− **168880** +15.56757·T+7·GCuFcc0+2·GZrHcp0
		GCu:ZrCu7Y20=− **55642** +7·GCuFcc0+2·GZrHcp0
		LCu:Y,ZrCu7Y20=− **35442**
CuY	(Cu)_1_(Y)_1_	GCu:YCuY0=− **44760** +4.12·T+GCuFcc0+GYHcp0

## Data Availability

The data that support the findings of this study are available from the corresponding author.

## Supplementary Materials

The following supporting information can be downloaded at: https://www.mdpi.com/article/10.3390/ma16052063/s1, Table S1. Summary of the crystallographic data for stable phases in the Cu–Zr–Y system. References [20,27,28,29,30,31,32,33,34,35] are cited in the supplementary materials.
