# Supplementary material for: Phase Equilibria, Thermodynamics and Solidified Microstructure in the Copper–Zirconium–Yttrium System
Source: Materials (Basel). 2023 Mar 2;16(5):2063. doi: 10.3390/ma16052063 (PMC10003912; doi:10.3390/ma16052063)
Supplement: Supplementary file 1 [file materials-16-02063-s001.zip › materials-2177736-supplementary.pdf]

Table S1. Summary of the crystallographic data for stable phases in the Cu–Zr–Y system

**Phase**	**Person symbol**	**Space group**	**Prototype**	**Lattice parameters, pm**			**Ref.**
Cu	*cF*4	$Fm\bar{3}m$	Cu	*a*=361.52			[1]
(αY)	*hP*2	*P*6$_3$/mmc	Mg	*a*=364.82		*c*=573.18	[2]
(βY)	*cI*2	$Im\bar{3}m$	W	*a*=407			[2]
(αZr)	*hP*2	*P*6$_3$/*mmc*	Mg	*a*=323.3		*c*=515.2	[3]
(βZr)	*cI*2	$Im\bar{3}m$	W	*a*=355.1			[4]
Cu_5Zr	*cF*24	$F\bar{4}3m$	$AuBe_5$	*a*=687			[5]
$Cu_{51}Zr_{14}$	*hP*68	*P*6/*m*	$Gd_{14}Ag_{51}$	*a*=112.4		*c*=828.2	[6]
Cu_8Zr_3	*oP*44	$F\bar{4}3m$	Hf_3Cu_8	*a*=786.9	*b*=998.5	*c*=815.5	[6]
$Cu_{10}Zr_7$	*Cmca*	*oS*68	Zr_7Ni_{10}	*a*=126.75	*b*=931.3	*c*=934.7	[7]
CuZr	*cP*2	$Pm\bar{3}m$	CsCl	*a*=326.2			[8]
$CuZr_2$	*tI6*	*I*4/*mmm*	$CuZr_2$	*a*=322		*c*=111.83	[9]
CuY	*cP*2	$Pm\bar{3}m$	CsCl	*a*=347.7			[10]
Cu_2Y_R		*hR**					[1]
Cu_2Y_H		*hP**					[1]
Cu_7Y_2	*hP*65	*P*6/*m*	$CeCu_{3.6}$	*a*=1156.9		*c*=867.4	[10]
Cu_4Y	*hp*6	*P*2/*m*	$CaCu_5$	*a*=500		*c*=410.5	[10]
Cu_6Y	*hP*8	*P*6/*mmm*	$TbCu_7$	*a*=496.8		*c*=412.3	[10]

References

1. Fries, S.G.; Lukas, H.L.; Konetzki, R.; Schmid-Fetzer, R. Experimental investigation and thermodynamic optimization of the Y-Cu binary system. *J. Phase Equilib.* **1994,** *15*, 606-614. https://doi.org/10.1007/BF02647621
2. Okamoto, H. Cu-Y (Copper-Yttrium). *J. Phase Equilib.* **1992,** *13*, 102-103.
3. Ellinger, F.H.; Land, C.C. On the plutonium-zirconium phase diagram. *Nucl. Metall.* **1971,** *17*, 686-698.
4. Yasohama, K.; Ogasawara, T. Specific heat and superconducting properties of zirconium-molybdenum alloys. *J. Phys.Soc. Japan* **1974,** *36*, 1349-1355.
5. Forey, P.; Glimois, J.L.; Feron, J.L.; Develey, G.; BECLE, C. Cheminform abstract: synthesis, characterization and crystal structure of copper-zirconium (Cu_5Zr). *Chemischer Informationsdienst* **1981,** *12*. https://doi.org/10.1002/chin.198108040

6. Bsenko, L. Crystallographic data for intermediate phases in the copper-zirconium and copper-hafnium systems. *J. Less-Common Met.* **1975,** *40*, 365-366.
7. Glimois, J.L.; Forey, P.; Feron, J.; Becle, C. Structural investigations of the pseudo-binary compounds nickel-copper-zirconium (Ni10-xCuxZr7). *J. Less-Common Met.* **1981,** *78*, 45-50.
8. Carvalho, E.M.; Harris, I.R. Constitutional and structural studies of the intermetallic phase, zirconium-copper (ZrCu). *J. Mater. Sci.* **1980,** *15*, 1224-1230.
9. Nevitt, M.V.; Downey, J.W. A family of intermediate phases having the Si_2Mo type structure. *Trans. Am. Inst. Min. Metall.Pet. Eng* **1962,** *224*, 195-196.
10. Belyavina, N.N.; Markiv, V.Y.; Nakonechna, O.I. Reinvestigation of the Y–Cu–Ga system at 700°C. *J. Alloys Compd.* **2012,** *541*, 288-296. https://doi.org/10.1016/j.jallcom.2012.07.034
